# Deregulation of Hexokinase II Is Associated with Glycolysis, Autophagy, and the Epithelial-Mesenchymal Transition in Tongue Squamous Cell Carcinoma under Hypoxia

**DOI:** 10.1155/2018/8480762

**Published:** 2018-02-21

**Authors:** Guanhui Chen, Yadong Zhang, Jianfeng Liang, Wenqing Li, Yue Zhu, Ming Zhang, Cheng Wang, Jinsong Hou

**Affiliations:** ^1^Department of Oral and Maxillofacial Surgery, Hospital of Stomatology, Guanghua School of Stomatology, Sun Yat-sen University, Guangzhou, Guangdong 510055, China; ^2^Guangdong Provincial Key Laboratory of Stomatology, Sun Yat-sen University, Guangzhou, Guangdong 510055, China

## Abstract

The glycolytic enzyme Hexokinase (HKII) participates in tumor glycolysis and the progression of various cancers, but its clinicopathological effect on the progression of tongue squamous cell carcinoma (TSCC) and its role in glycolysis, autophagy, and the epithelial-mesenchymal transition of TSCC in a hypoxic microenvironment remain unknown. Our results showed that HKII expression was dramatically increased in TSCC tissues and that its upregulation was significantly associated with the presence of pathological differentiation, lymph node metastasis, and clinical stage. The level of autophagy-specific protein LC3, EMT-related proteins, and the migration and invasion capabilities of TSCC cells all increased under hypoxia. Moreover, hypoxia increased the glucose consumption and lactate production of TSCC cells, and we demonstrated that the expression of the glycolytic key gene HKII was significantly higher than in that of the control group. Notably, the downregulation of HKII resulted in a significant decrease of TSCC cell glucose consumption lactate production and autophagic activity during hypoxia. HKII knockdown blocked the migratory and invasive capacity of TSCC cells and we specifically determined that the EMT ability decreased. Therefore, our findings revealed that the upregulation of HKII enhanced glycolysis and increased autophagy and the epithelial-mesenchymal transition of tongue squamous cell carcinoma under hypoxia.

## 1. Introduction

Tongue squamous cell carcinoma (TSCC), the most common type of oral malignant tumor, is characterized by a highly aggressive potential [1]. Despite the advanced therapeutic strategies that have been developed in the past several decades, the overall survival rate of TSCC patients still remains poor due to high rates of local invasion and metastasis [2]. Increasing evidence has shown that a hypoxic tumor microenvironment due to poor vascularization is closely associated with tumor migration and invasion [3]. However, the mechanisms by which tumor cells maintain a high rate of growth and whether the migratory and invasive capacity of TSCC is altered under hypoxic conditions are still poorly understood.

The Warburg effect describes a form of abnormal metabolism in which cancer cells preferentially use glycolysis for energy, producing lactate as an end product, despite being in the presence of oxygen [4]. Hexokinase II (HKII) is the first main rate limiting enzyme that regulates the glycolytic rate to promote a tumor cell's survival. Most cancer tissues express a high level of HKII, which is associated with prognosis [5, 6]. However, to the best of our knowledge, the relationship between HKII expression and TSCC progression, as well as whether HKII-mediated glycolytic flux alters when TSCC cells are under hypoxia, has not been fully elucidated.

Autophagy, which has emerged as the major lysosomal pathway for recycling intracellular components, including damaged organelles and misfolded proteins, is one of the main mechanisms by which tumor cells adapt to an adverse tumor microenvironment [7]. Studies have confirmed that autophagy can be activated under conditions of hypoxia and participates in the promotion of tumor development [8]. However, methods to alter autophagic activity of TSCC have not been documented when the cells suffer from hypoxia. The epithelial-mesenchymal transition (EMT) defines the phenotypic switch from epithelial cells into a mesenchymal-like phenotype, leading to morphological changes to the fibroblastoid morphology [9]. Increasing evidence has indicated that EMT is a crucial mechanism mediating tumor metastasis [10]. Li et al. discovered that autophagy promoted hepatocellular carcinoma invasion through the activation of the epithelial-mesenchymal transition [11]. However, whether EMT is involved in hypoxia-induced autophagy remains poorly understood in TSCC.

In this study, we first aimed to characterize the roles of autophagy, glycolysis, EMT, migration, and invasion in TSCC cells under hypoxia and to identify the key metabolic molecule modulating autophagy and metastasis. Here, our results reveal that increased HKII expression was closely linked to tumor stage, pathological differentiation, lymph node metastasis, and clinical stage. Hypoxia-induced autophagy promoted the glycolysis of TSCC cells by targeting the glycolytic key enzyme HKII; HKII inhibition obviously blocked the metastatic potential and EMT ability of TSCC. This research establishes a strong relationship between autophagy, glycolysis, and the malignant phenotype of TSCC cells under hypoxic conditions, which might represent a powerful approach for the development of novel TSCC therapy.

## 2. Materials and Methods

### 2.1. Patients and Tissue Specimens

All tissue samples were collected from the First Affiliated Hospital of Sun Yat-sen University between September 2010 and October 2015. In total, 95 TSCC patients who had received radical therapy without previous surgery, radiotherapy, or chemotherapy and 20 matching adjacent noncancerous tissues (ANTs) were obtained for HKII immunohistochemical assessment. All patients gave informed consent for research purposes. This study was approved by the Institution of the Hospital of Stomatology of Sun Yat-sen University for ethical integrity.

### 2.2. Immunohistochemical Assay

Formalin-fixed, paraffin-embedded tissues were cut into 3.5 *μ*m sections and processed for immunostaining. Briefly, the sections were dewaxed, rehydrated, and blocked at room temperature, whereupon the slides were blocked with 10% goat serum followed by antigen retrieval. Tissues sections were incubated with an antibody against HKII (1 : 200, Abcam) at 4°C overnight. After incubation in horseradish peroxidase, the slides were visualized using diaminobenzidine (DAB, Sigma-Aldrich) and counterstained with hematoxylin. The degree of immunostaining was scored by the proportion of positive cells (0, 0%; 1, <25%; 2, <50%; 3, <75%; 4, ≥75%) and staining intensity (0, no staining; 1, weak; 2, moderate; 3, strong) and the final HKII expression was determined by staining index (staining intensity score × proportion of positive cells). Cases with a staining index > 4 were defined in the high-expression group, while a score ≤ 4 classified cases as demonstrating low HKII expression.

### 2.3. Cell Culture

Human tongue squamous cell carcinoma cell lines UM1, SCC15, and SCC25 were cultured in Dulbecco's modified Eagle's medium/Ham's F12 (Gibco, USA) supplemented with 10% fetal bovine serum (Gibco, USA) and 400 ng/ml Glucocorticoid (Sigma-Aldrich, USA). All cell lines were grown in humidified conditions with 5% CO_2_ at 37°C, while hypoxia treatment groups were maintained in a hypoxic incubator chamber (Eppendorf, Germany) containing 1% O_2_ and 94% N_2_ for various amounts of time under hypoxia.

### 2.4. Western Blotting

All cellular proteins were extracted with RIPA buffer, with the addition of 0.1% PMSF (Cwbio, China). The supernatant was collected by centrifugation at 14000*g* for 25 min at 4°C and the BCA protein assay kit (Cwbio, China) was used to measure the protein concentration, according to the manufacturer's instructions. Equal amounts of protein (25 ug) were then separated by electrophoresis on a SDS-PAGE gel and electrotransferred to PVDF membranes (Millipore, USA). After blocking with 5% bovine serum albumin (Cwbio, China) for 1 h, immunodetection was performed with primary antibodies, including Beclin-1, LC3, HIF-1*α*, HKII, PFKP, PKM2, Glut-1, E-cadherin, Vimentin, Snail, and Slug (Cell Signaling, USA). All bands were probed with GAPDH or *β*-actin antibodies as the control. After incubation with the corresponding secondary antibody, the target proteins were visualized using an ECL kit (Millipore, USA) and quantified with Image-J software analysis.

### 2.5. Real-Time Polymerase Chain Reaction

RNA was isolated using the RNA extraction kit (Molecular Research Center, USA) and was reverse transcribed to cDNA using a cDNA synthesis kit (TaKaRa, Japan) following the manufacturer's instructions. The quantitative real-time PCR was performed for HKII on the LightCycler 480 System with SYBR Green I Master Mix Reagent kit (Roche, Germany) according to the standard protocol. The following sequences of the HKII and GAPDH used are as follows (HKII: Forward: 5′-CCTTCTCCCCTTCAATGTCTG-3′, Reverse: 5′-CTGGTGAGGCTTATCCTGGTG-3; GAPDH: Forward: 5′-TCTCCTCTGACTTCAACAGCGACA-3′, Reverse: 5′-CCCTGTTGCTGTAGCCAAATTCGT-3′). The mRNA level fold changes were normalized to those of GAPDH and the relative expression level of HKII was finally calculated using the 2^−ΔΔCt^ method.

### 2.6. RNA Transient Transfection

All cells were transfected with control or HKII siRNA using a transfection kit (RiboBio, China) according to the manufacturer's protocols. In short, siRNAs were dissolved in the supplied buffer for 5 min, and then the lipofectamine RNAimax regents (Thermo Fisher, USA) were mixed with the siRNA-buffer solution for 10 min. Finally, the compounds were added to the culture medium and with a final siRNA concentration of 50 nM. The cells were then incubated in a 37°C incubator containing 5% CO_2_. After transfection for 24 h, the three different sequences and the negative control sequence were confirmed, in order to test their expression by RT-qPCR and Western blot for optimal sequence knockdown. Next, the experimental groups were maintained in a hypoxic incubator chamber for 12 h after transfection. The control groups were cultured in a humidified atmosphere of 5% CO_2_ and 95% air. The three independent HKII siRNA target sequences were as follows: siHKII01: CTGTGAAGTTGGCCTCATT; siHKII02: ACGACAGCATCATTGTTAA; and siHKII03: CTGGCTAACTTCATGGATA.

### 2.7. Glucose Consumption and Lactate Production Assay

All cells were seeded in 6-well plates at a density of 3.0 × 10^5^ cells per well in 2 ml DMEM/F12 medium. A blank well without cells was used as the control. Cells were cultured in completed medium of either normal or hypoxia groups. After 3, 6, or 9 h incubation, glucose and lactate concentrations in the supernatant medium were determined using a commercial kit (glucose assay kit, lactate assay kit; Nanjing Jiancheng Biotech, China) in accordance with the manufacturer's instructions. The glucose consumption and lactate production for each group were computed as the difference between the blank medium and the sample medium. Values were compared with cell numbers and the final results were expressed as pmol per 1.0 × 10^5^ cells.

### 2.8. Transwell Assay

The ability of cell migration and invasion were detected using the BD Biocoat system (BD Biosciences, USA). Briefly, for the invasion assay, the membrane surface was coated with matrigel and 8 × 10^4^ cells were seeded in the upper chambers and suspended with DMEM/F12 medium without FBS, while the lower chambers were immersed in complete medium. Cells from the normal group were incubated at 37°C, 5% CO_2_, and 95% air, whereas hypoxia group cells were maintained in a hypoxic incubator chamber. After 12 h invasion, the matrigel and cells on the top surface of the chamber were eliminated using a cotton swab. Cells adhering to the reverse side of the inserts were fixed in 4% paraformaldehyde for 25 min and stained with 0.1% crystal violet solution for 5 min. Cells were counted in five randomly selected views using an inverted microscope. For the migration assay, the procedure was similar to that of cell invasion, except that the membranes did not have matrigel. All assays were independently repeated in triplicate.

### 2.9. Statistical Analysis

All values are reported as mean ± SD. Statistical analysis was performed using SPSS version 19.0 (Chicago, USA) and the Student's *t*-test or ANOVA was used for comparisons between groups. The correlation between HKII expressions and the clinicopathological characteristics of TSCC patients were assessed using Fisher's exact test. Differences were considered to be statistically significant when *P* < 0.05.

## 3. Results

### 3.1. HKII Is Upregulated in TSCCs

**Figure 1:**
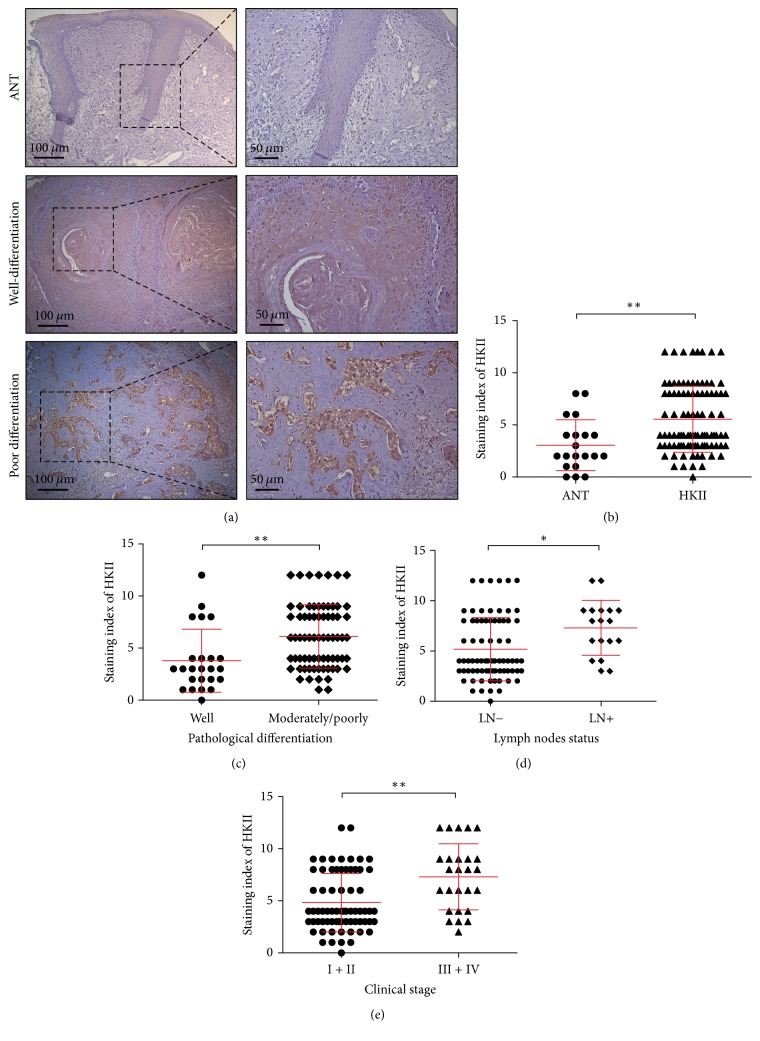
HKII is upregulated in TSCC. (a) Representative immunohistochemistry images for HKII staining in adjacent noncancerous tissues (ANT) and TSCC samples including well-differentiated (*n* = 24) and moderately/poorly differentiated samples (*n* = 71). Magnification 100x (left panel) and 200x (right panel). (b–e) Vertical scatter plots demonstrate the relative levels of HKII expression in ANTs and TSCCs (b), TSCC with different pathological differentiation (c), lymph node metastasis (d), and clinical stages (e). ^*∗*^*P* < 0.05, ^*∗∗*^*P* < 0.01.

In order to investigate the role of HKII progression, we performed an immunohistochemical analysis of the HKII levels in the TSCC specimens obtained from 95 TSCC patients and 20 patients with adjacent noncancerous tissues (ANT). The ANT of TSCC patients showed lower HKII levels than did either their counterparts in the well-differentiated or poorly differentiated cancerous groups (Figure 1(a)). The average expression of HKII significantly increased in TSCC specimens compared with ANT (Figure 1(b)). Furthermore, in order to examine the clinicopathological significance of HKII level in patients with TSCC, a correlation analysis of immunostaining index demonstrated that HKII expression was closely associated with pathological differentiation (Figure 1(c)), lymph node metastasis (Figure 1(d)), and clinical stage (Figure 1(e)); all TSCC tissue groups had statistically significant difference. However, no significant differences were identified between HKII expression and age, gender, or T grade (Table 1). Thus, we suggest that HKII expression correlates with the development and progression of TSCC.

### 3.2. Hypoxia Induced Higher HKII Expression and Enhanced the Glycolytic Rate in TSCC Cells

**Figure 2:**
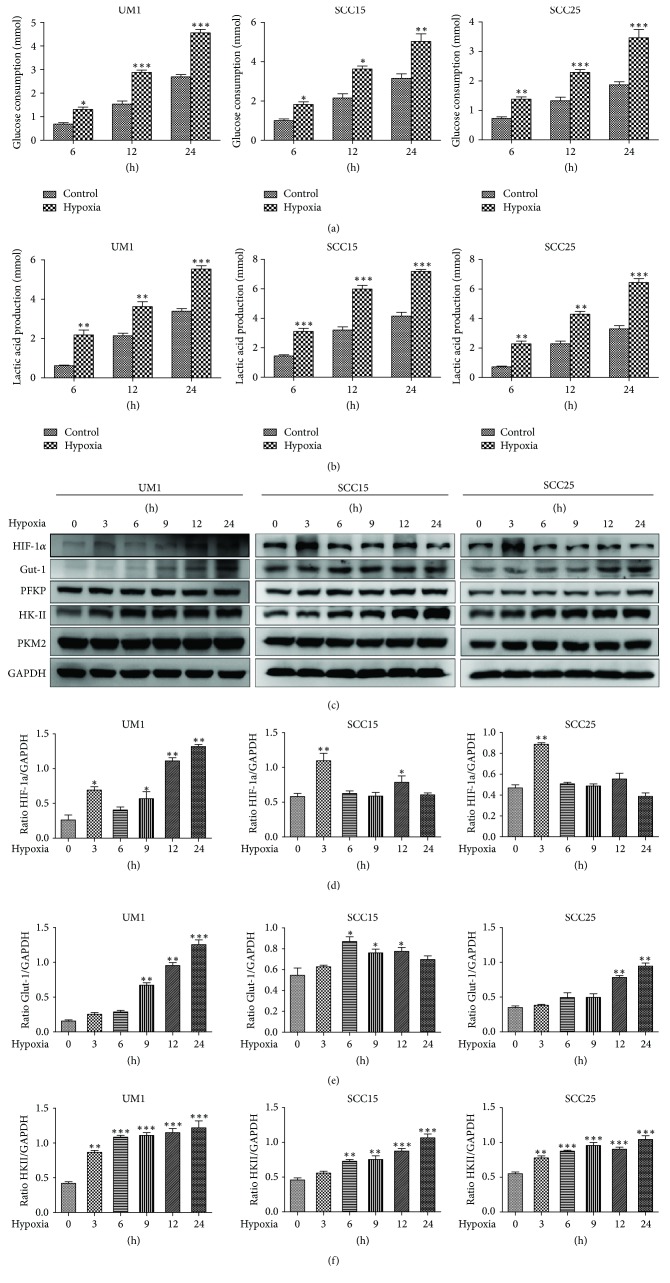
Effects of hypoxia on the glycolysis of TSCC cells. (a) Glucose consumption in TSCC cells significantly increased under hypoxia for different periods of time. (b) Lactate production in TSCC cells dramatically increased under hypoxia. (c) Hypoxia induced higher expression of HIF-1*α*, Glut-1, and HKII in all three cell types. (d) The HIF-1*α* levels were estimated as a ratio with the levels of GAPDH for all three cell lines. (e) The quantitative Glut-1 data was shown as a ratio with the levels of GAPDH in TSCC cells. (f) The quantitative HKII analysis was presented after normalization to GAPDH in TSCC cells. All results are presented as mean ± SD obtained from three independent experiments. ^*∗*^*P* < 0.05, ^*∗∗*^*P* < 0.01, and ^*∗∗∗*^*P* < 0.001.

Considering that tumor microenvironments are often hypoxic, we set out to determine whether the glycolytic rate was expedited to provide more energy to adapt to the unfavorable microenvironment. In order to test our hypothesis, glucose consumption and lactate production were measured in the culture supernatants of all three cell lines during different lengths of hypoxia. The results showed dramatic increases in both glucose consumption and lactate production in UM1, SCC15, and SCC25 cells under hypoxia (Figures 2(a) and 2(b)). Moreover, HIF-1*α*, an important transcription factor that regulates multiple target genes [12], was remarkably higher in TSCC cells after 3 h hypoxia (Figures 2(c) and 2(d)). To further explore the mechanism of glycolysis enhancement, we analyzed whether or not Glut-1, which is more pervasive in tumors, and three glycolytic rate limiting enzymes (HKII, PFKP, and PKM2) would be altered when cells suffered from hypoxia, as assessed by western blot. The results showed that Glut-1 and HKII were significantly enhanced in all three cell types, yet the other two limiting enzymes PFKP and PKM2 showed no obvious change under hypoxia (Figures 2(c) and 2(e)-2(f)). These results suggest that hypoxia induced higher HKII expression, which in turn might have contributed to the acceleration of aerobic glycolysis in TSCC cells.

### 3.3. Hypoxic Conditions Induced Autophagy and Promoted the Migration and Invasion Involved in the Enhancement of EMT in TSCC Cells

**Figure 3:**
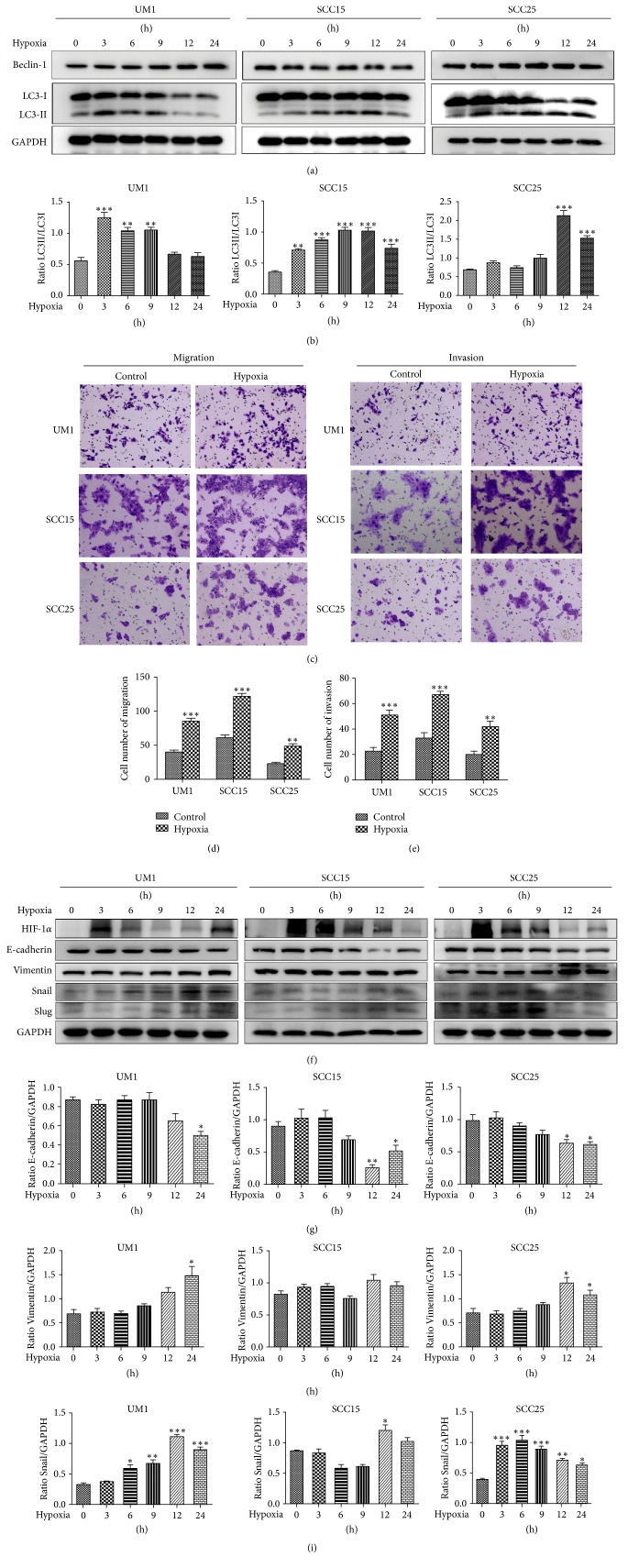
Hypoxia induces autophagy and enhances the migration and invasion of TSCC cells. (a) The expressions of Beclin-1 and LC3 were confirmed by western blot. (b) The level of the LC3 protein was estimated as a ratio of LC3-II to LC3-I. (c–e) A transwell assay was performed to measure cell metastasis (c) and migration (d) and invasion (e) were both quantified. Images are presented at 100x magnification. (f) HIF-1*α*, E-cadherin, Vimentin, Snail, and Slug levels of cells from all three cell lines were evaluated by western blot. (g–i) Quantitative data for E-cadherin (g), Vimentin (h), and Snail (i) are shown as a ratio to the GAPDH level in TSCC cells. All values are shown as mean ± SD of triplicate experiments. ^*∗*^*P* < 0.05, ^*∗∗*^*P* < 0.01, and ^*∗∗∗*^*P* < 0.001.

To determine the effect of hypoxia on autophagy and malignant phenotype in TSCC cells, western blot was initially conducted to examine the autophagy-related gene levels of UM1, SCC15, and SCC25 cells at different time points. Compared with the normoxic group, the autophagy-specific marker LC3-II was significantly upregulated at various time points in all three cell lines, and the autophagy-related protein Beclin-1 was increased in UM1 and SCC25 cells but showed no change in SCC15 cells after hypoxia (Figure 3(a)). The levels of LC3 were also summarized (Figure 3(b)), and the results suggested that hypoxia enhanced the autophagic activity of TSCC cells. Next, a transwell assay was performed to examine the biological role of hypoxia on the migration and invasion of TSCC cells. The results showed the migratory and invasive capacity of cells in all hypoxia groups were greater than those of the control cells (Figures 3(c)–3(e)). Furthermore, in order to probe the mechanisms by which hypoxia promotes TSCC cell metastasis, western blot illustrated that the expression level of HIF-1*α* increased under hypoxia and that the expression of the epithelial-mesenchymal transition protein E-cadherin was decreased in all three cell lines until after 12 h of hypoxia (Figures 3(f) and 3(g)). The Vimentin level was upregulated in UM1 and SCC25 cells and was not altered in SCC15 cells, but the expressions of the transcription factors Snail and Slug increased and we summarized the levels of Vimentin and Snail in all three cell lines during hypoxia (Figures 3(h) and 3(i)). These findings revealed that hypoxia promoted the cell migration and invasive capacities of TSCC with consistent enhancement of EMT activity.

### 3.4. Silencing HKII Leads to the Suppression of Glycolysis in TSCC Cells

**Figure 4:**
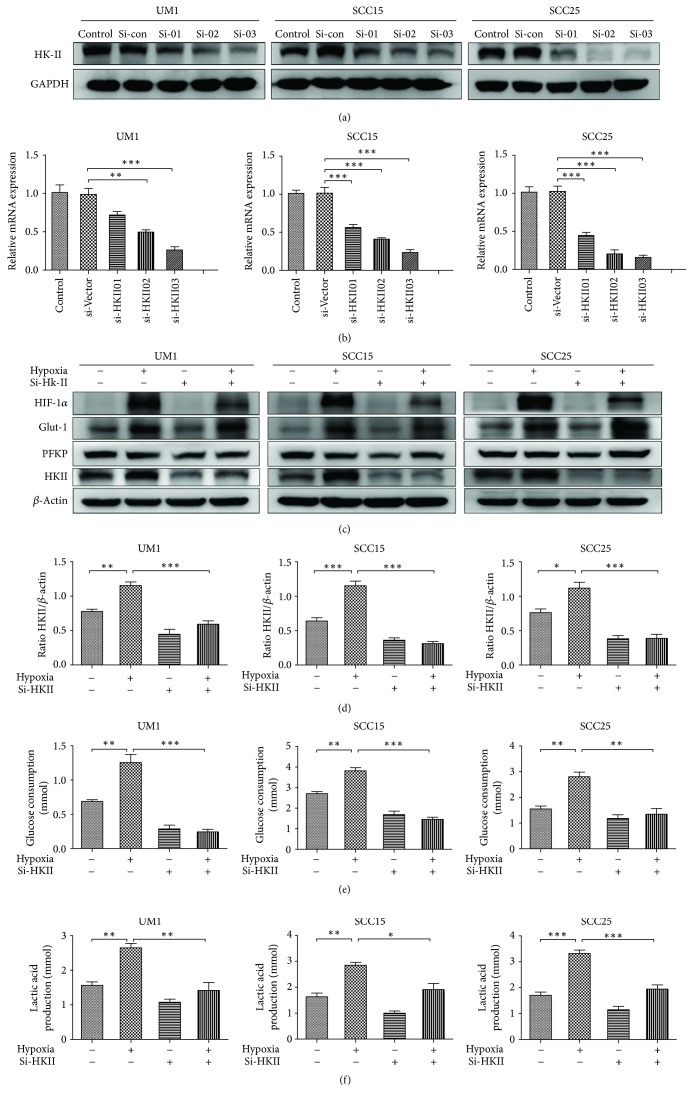
HKII knockdown inhibited glycolysis in TSCC cells. (a) The HKII levels were examined by western blot after 24 h transfection. (b) The expression of HKII for transient transfection of TSCC cells was detected by RT-PCR. (c) The levels of HIF-1*α*, Glut-1, PFKP, and HKII were measured with western blot after transfection of TSCC cells. (d) Quantitative analysis of HKII was presented. (e) Representative results of glucose consumption were significantly decreased after suppression of HKII. (f) Lactate production was obviously reduced after suppression of HKII. Data are presented as mean ± SD of three experiments. ^*∗*^*P* < 0.05, ^*∗∗*^*P* < 0.01, and ^*∗∗∗*^*P* < 0.001.

As previously identified, the expression of HKII was higher in TSCC cell lines under hypoxia, indicating a higher glycolytic activity in TSCC cells. In order to further investigate the role of HKII suppression on TSCC cells, three different HKII siRNAs were used to decrease HK2 expression in all three cell lines for selecting the optimal knockdown sequence by western blot and RT-PCR (Figures 4(a) and 4(b)). The knockdown of si-HKII03 markedly inhibited HKII activity in all three cell lines, whereupon it was used for follow-up experiments. Interestingly, we found that the levels of Glut-1 did not obviously alter after HKII suppression; however it was significantly improved correspondingly with the increase in HIF-1*α* as described before, suggesting that its expression was dependent on the induction of HIF-1*α* (Figures 4(c) and 4(d)). Next, we tested whether the glycolytic activity of all three cell types would be altered by measuring glucose consumption and lactate production after the inhibition of HKII. As expected, HKII knockdown clearly resulted in reduced glucose consumption and lactate production after hypoxia treatment (Figures 4(e) and 4(f)). All of the above results suggest that silencing HKII inhibited glycolysis metabolism in TSCC cells.

### 3.5. The Inhibition of HKII Downregulated Autophagy and Attenuated the Migration, Invasion, and EMT Ability of TSCC Cells

**Figure 5:**
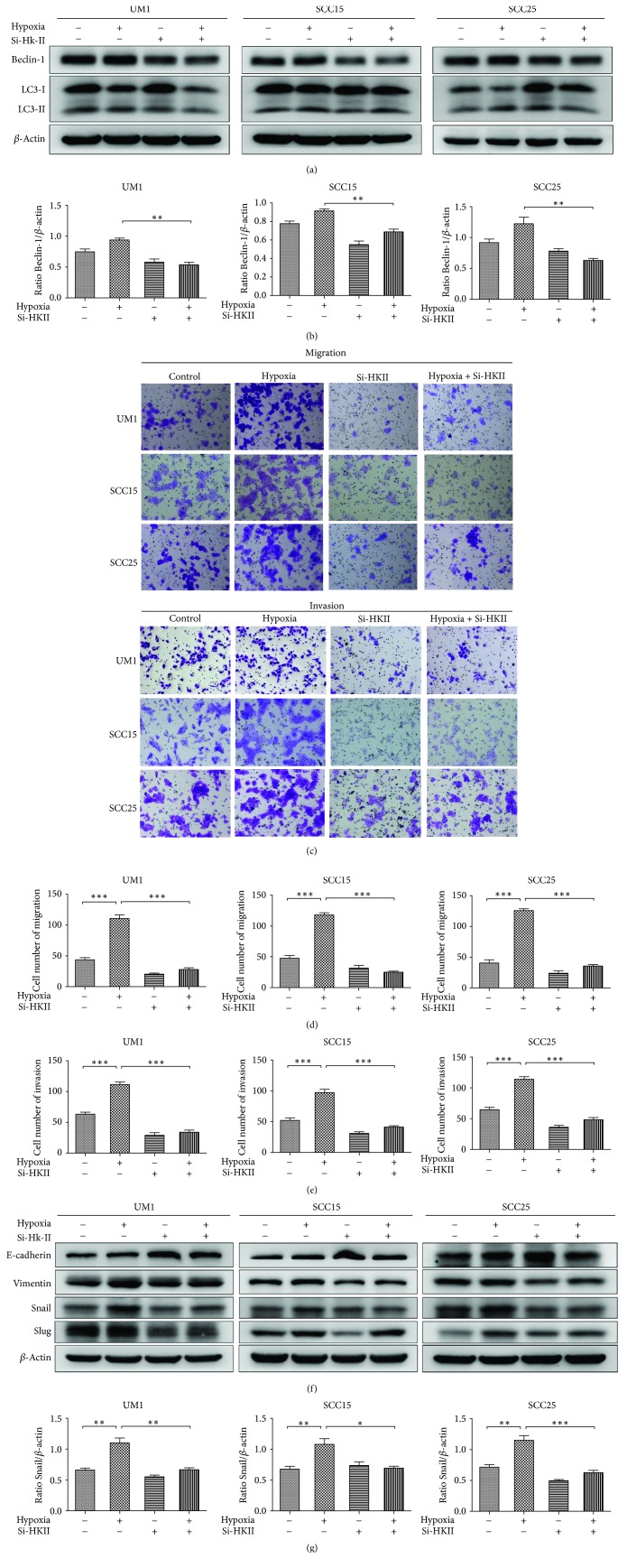
Effects of HKII knockdown on the ability of autophagy, metastasis, and EMT in TSCC cells. (a) The levels of Beclin-1 and LC3 were evaluated after transient transfection. (b) The quantitative Beclin-1 data were computed as a ratio to levels of *β*-actin. (c) Representative results of the migration and invasion ability of TSCC cells were evaluated after transfection. Representative pictures of ×100 magnification. (d) Quantitative analysis of migratory ability. (e) The invasive ability quantitative data. (f) E-cadherin, Vimentin, Snail, and Slug expression levels were assessed after 24 h transfection. (g) Quantitative Snail data presented as a ratio to the *β*-actin level. All results are presented as mean ± SD obtained from three independent experiments. ^*∗*^*P* < 0.05, ^*∗∗*^*P* < 0.01, and ^*∗∗∗*^*P* < 0.001.

Beclin-1, an early autophagy-related gene, and LC3 are the hallmarks of autophagy induction [13]. In order to investigate the effects of HKII inhibition on autophagy, the expression levels of autophagy-related genes were observed by western blot. The results demonstrated that HKII knockdown decreased Beclin-1 and LC3-II levels following treatment with hypoxia + si-HKII as compared with the hypoxia group in all three cell lines (Figures 5(a) and 5(b)). These results suggest that the suppression of HKII decreased autophagic activity in response to hypoxia in TSCC cells. Furthermore, to validate the role of HKII inhibition on the migratory and invasive capacity of TSCC cells after 24 h transfection, the transwell assay was used and the results found that the hypoxia + si-HKII groups had severely impaired migration and invasion capacities relative to the hypoxia group (Figures 5(c)–5(e)). Moreover, in order to explore the mechanistic link between HKII and the malignant phenotype of TSCC cells, we detected the expression of EMT markers using western blot. The results showed that the Vimentin and Snail levels were reduced in all three cell lines transfected with si-HKII when compared with the control groups under hypoxia, while the expressions of E-cadherin and Slug were correspondingly altered in the UM1 and SCC25 cell lines (Figures 5(f) and 5(g)). Taken together, these results reveal that EMT was involved in the HKII-mediated promotion of migration and invasion in TSCC cells.

## 4. Discussion

HK is the first key glycolytic enzyme that irreversibly catalyzes the formation of glucose-6-phosphate from glucose and thereby contributes to cellular protection against stress [14, 15]. It is now known that four isoforms of HK have been confirmed, among which HKII is highly expressed in most cancers [16]. Wolf et al. discovered that the overexpression of HKII can promote tumor growth in human glioblastoma multiforme [17]. Moreover, low oxygen tension is a common characteristic of solid tumors during their rapid proliferation and plays a vital role in tumor progression [18]. Studies have confirmed that concentrations of oxygen lower than 3% are considered a hypoxic environment [19]. Hypoxia-inducible factor-1*α* (HIF-1*α*) is characterized by a short half-life and unstable expression and is a key transcription factor that binds hypoxia-responsive element (HRE) in the promoters of target genes, resulting in the activation of transcription [20], and was clearly increased at an oxygen concentration of <1% oxygen. In our present study, HKII was significantly increased in TSCC samples and its expression was meaningfully related to pathological differentiation, lymph node metastasis, and clinical stage in TSCC. GAPDH was used as a housekeeping gene in this research. Although previous contributions showed that GAPDH expression was regulated by hypoxia, other studies indicated that there was no relationship between the expression of GAPDH and hypoxia [21, 22]. Our data also revealed that there was no hypoxia-dependent regulation of GAPDH in TSCC cells. Then, we evaluated the three main glycolytic key enzymes and found that the levels of PFKP and PKM2 have no apparent change; however the expression of HKII was increased under hypoxic conditions. Further study confirmed that HKII is also the target gene of HIF-1*α*, which can directly promote the transcriptional expression of HKII under hypoxia [23]. This was consistent with our experimental results.

The “Warburg effect,” a phenomenon of preferred aerobic glycolysis that is thought to be the major metabolic approach of tumor cells, is closely related to tumor metastasis [24]. In this study, we demonstrated that the glucose consumption and lactate production were significantly increased under hypoxic conditions, indicating that the glycolytic activity of TSCC cells was upregulated. Glut-1, a major glucose transporter, was confirmed as the target gene of HIF-1*α* [25]. Consistent with our results, the hypoxia-induced upregulation of HIF-1*α* resulted in elevated levels of Glut-1 in TSCC cells, further proving that Glut-1 activity was regulated by HIF-1*α*.

Autophagy, a conserved dynamic pathway, protects cells from hypoxia stress by replenishing energy metabolites and maintaining viability [26]. Research has confirmed that the autophagic ability of most tumors increases under hypoxic conditions [27]. Viry et al. demonstrated that HIF-1*α* combined with HRE2 on the BNIP3 protein, which induced autophagy through the activation of the PI3K signaling pathway [28]. Furthermore, Thongchot et al. identified that hypoxia-induced autophagy promoted cholangiocarcinoma cell survival and metastasis [29]. Similarly, in this study, we first illustrated that the autophagic activity of TSCC was increased by the enhancement of the ratio of the autophagy-related protein LC3-II/LC3-I under hypoxia. Additionally, in order to detect change in the TSCC malignant phenotype under hypoxia using transwell assays, we explored the way in which hypoxia-induced autophagy promoted migration and invasion in TSCC, but the underlying mechanisms are still poorly understood. EMT, a crucial mechanism by which epithelial cells lose adhesive properties and acquire the mesenchymal phenotype, initiates the metastatic behavior of a tumor [30]. Jiang et al. indicated that HIF-1*α* acts together with TGF-*β* to induce EMT by phosphorylating SMAD, leading to the promotion of cancer progression [31]. To further confirm the effect of EMT on migration and invasion in TSCC cells, our results demonstrated that hypoxia induced the decreased expression of E-cadherin and the increased expression of Vimentin, Snail, and Slug, suggesting that the improved EMT ability was involved in the migration and invasion of TSCC cells under hypoxia-induced autophagy.

Studies have shown that silencing HKII could inhibit glycolysis and reduce the proliferation of tumor cells [32]. However, the role of HKII in regulating TSCC metabolism, migration, and invasion is not well known under conditions of hypoxia. We found that the downregulation of HKII could reduce HIF-1*α* levels. Some research has identified that the loss of HKII led to a reduced activation of ERK phosphorylation in tumor cells, and other studies indicated that the activation of ERK can promote the HIF-1*α* translation by phosphorylating MAP kinase interacting kinase (MNK) [33, 34]. Thus, this mechanism might be associated with ERK signaling, which should be verified in future studies. Furthermore, we confirmed that HKII knockdown decreased glucose consumption and lactate production in TSCC cell lines, indicating inhibited glycolysis. In addition, the downregulation of HKII also inhibited cellular migratory and invasive capacities, demonstrating that HKII modulates the metastatic ability of TSCC cells. Meanwhile, HKII knockdown also weakened the levels of Beclin-1 and LC3-II in response to hypoxia, supporting the observation that the ability of autophagy was inhibited. Zhuo et al. showed that the expression of HKII was associated with the activation of the PI3K/AKT signaling pathway, which promoted the development of pediatric osteosarcoma [35]. However, the PI3K/AKT signaling pathway is also closely related to AMPK activity. Another study indicated that AMPK activation promoted the transcriptional expression of the HKII gene in rat skeletal muscle [36]. We speculate that the inhibition of HKII and resulting reduction in autophagic ability might be correlated with the inactivation of the AMPK protein under the hypoxia model. Nevertheless, the exact mechanism of AMPK on HKII knockdown is still unclarified and requires future investigations.

Given the fact that autophagy provides a survival advantage to tumor cells suffering from hypoxic stress [37], we verified the interaction of HKII knockdown on the migratory and invasive behavior of TSCC cells. Here, we suggest that the knockdown of HKII resulted in the attenuation of the malignant phenotype, which might be closely linked with decreased autophagy activity. On the other hand, the reduction in the TSCC metastatic capacity also reflected the lower energy metabolism utilization, proving that the inhibition of glycolysis suppressed the principal pathway of tumor metabolism, which was consistent with the Warburg effect [38]. Still, in order to further elucidate the underlying mechanisms between HKII and molecules related to migration and invasion, we examined EMT-related proteins and found that the EMT ability was reduced when HKII was inhibited under hypoxia. All of the above results suggest that the HKII-mediated inhibition of migration and invasion may involve the suppression of EMT under hypoxia in TSCC cells.

## 5. Conclusions

Collectively, our work demonstrates the first implication that the glycolytic gene HKII is increased during TSCC progression and promotes the autophagy and metastatic potential of TSCC cells via upregulating glycolysis under hypoxia. These findings provide an experimental basis for the idea that inhibiting HKII may serve as a potential therapeutic target for TSCC.

## Figures and Tables

**Table 1:** Association between HKII expression and clinicopathological features in patients with OSCC (*n* = 95).

Clinicopathological features	Number of cases	HKII expression		*P* value^*∗*^
		High (*n*)	Low (*n*)	
Gender				
Male	56	29	27	0.843
Female	39	21	18	
Age				
≥55	62	32	30	0.785
<55	33	18	15	
Differentiation				
Well	24	5	19	**0.001 **
Moderate + poor	71	45	26	
T stage				
T_I-II_	84	43	41	0.437
T_III-IV_	11	7	4	
Clinical stage				
I-II	69	31	38	**0.014 **
III-IV	26	19	7	
LN metastasis				
N^−^	77	36	41	**0.018 **
N^+^	18	14	4	

^*∗*^ *P* < 0.05.
